# Kv4.3-Encoded Fast Transient Outward Current Is Presented in Kv4.2 Knockout Mouse Cardiomyocytes

**DOI:** 10.1371/journal.pone.0133274

**Published:** 2015-07-21

**Authors:** Jie Liu, Kyoung-Han Kim, Michael J. Morales, Scott P. Heximer, Chi-chung Hui, Peter H. Backx

**Affiliations:** 1 The Departments of Physiology and Medicine, University of Toronto, Toronto, Ontario, Canada; 2 Division of Cardiology, University Health Network, Toronto, Ontario, Canada; 3 Program in Developmental and Stem Cell Biology, Hospital for Sick Children, Toronto, Ontario, Canada; 4 Department of Physiology & Biophysics, University at Buffalo, the State University of New York, Buffalo, New York, United States of America; 5 The Departments of Molecular Genetics, University of Toronto, Toronto, Ontario, Canada; Brigham & Women's Hospital—Harvard Medical School, UNITED STATES

## Abstract

Gradients of the fast transient outward K^+^ current (I_to,f_) contribute to heterogeneity of ventricular repolarization in a number of species. Cardiac I_to,f_ levels and gradients change notably with heart disease. Human cardiac I_to,f_ appears to be encoded by the Kv4.3 pore-forming α-subunit plus the auxiliary KChIP2 β-subunit while mouse cardiac I_to,f_ requires Kv4.2 and Kv4.3 α-subunits plus KChIP2. Regional differences in cardiac I_to,f_ are associated with expression differences in Kv4.2 and KChIP2. Although I_to,f_ was reported to be absent in mouse ventricular cardiomyocytes lacking the Kv4.2 gene (Kv4.2-/-) when short depolarizing voltage pulses were used to activate voltage-gated K^+^ currents, in the present study, we showed that the use of long depolarization steps revealed a heteropodatoxin-sensitive I_to,f_ (at ~40% of the wild-type levels). Immunohistological studies further demonstrated membrane expression of Kv4.3 in Kv4.2-/- cardiomyocytes. Transmural I_to,f_ gradients across the left ventricular wall were reduced by ~3.5-fold in Kv4.2-/- heart, compared to wild-type. The I_to,f_ gradient in Kv4.2-/- hearts was associated with gradients in KChIP2 mRNA expression while in wild-type there was also a gradient in Kv4.2 expression. In conclusion, we found that Kv4.3-based I_to,f_ exists in the absence of Kv4.2, although with a reduced transmural gradient. Kv4.2-/- mice may be a useful animal model for studying Kv4.3-based I_to,f_ as observed in humans.

## Introduction

The fast transient outward potassium current (I_to,f_) modulates action potential profile, Ca^2+^ handling, contractility and hypertrophy in cardiomyocytes [1, 2]. I_to,f_ is also developmentally regulated [3] and its levels are invariably reduced in heart disease. Regional differences in I_to,f_ are major determinants of the transmural gradient of ventricular repolarization in many species [4, 5] and these gradients are often altered in diseased hearts [6]. The pore-forming α-subunits of I_to,f_ channels are comprised of Kv4.2 and Kv4.3 in rodents [7], but apparently only Kv4.3 in humans [8]. In both case, they form oligomeric complexes with the obligatory auxiliary subunit KChIP2, as well as either DPP6 or DPP10 [7]. While the I_to,f_ gradient in humans and dogs are linked to regional differences in KChIP2 expression [4, 9], the basis for regional differences in mouse I_to,f_ is not clear with studies concluding that either Kv4.2 or KChIP2 or both underlie the transmural gradient [10].

In rodents, the molecular basis of I_to,f_ is less clear. As I_to,f_ was undetected in mice lacking the Kv4.2 gene, a previous report concluded that the native cardiac I_to,f_ requires obligatory heterotetramers of Kv4.2 and Kv4.3 [11], even though I_to,f_ currents are present in mice lacking Kv4.3 [12] and can be generated in heterologous expression systems by either Kv4.2 or Kv4.3 α-subunits alone [5]. While several molecular explanations could account for these conflicting findings, the previous studies [11, 12] used short depolarization pulses (lasting ~5 sec) to quantify I_to,f_, which do not allow accurate dissection and quantification of K^+^ currents in mouse cardiomyocytes [13, 14]. Recently, we showed that long depolarization pulses (lasting ~25 sec) are required to accurately dissect and quantify the different K^+^ currents in mouse cardiomyocytes [12]. Therefore, we re-examined the existence of I_to,f_ in mice lacking the Kv4.2 gene (Kv4.2-/-) and found clear evidence for the presence of I_to,f_ in Kv4.2-/- hearts with a markedly reduced transmural gradient.

## Materials and Methods

### Transgenic animals and genotyping

All animal experiments were performed in accordance with protocols approved by the Faculty of Medicine and Pharmacy Animal Care Committee, University of Toronto, and conformed to the standards of the Canadian Council on Animal Care. Mice lacking of Kv4.2 gene (K_V_4.2-/-) [11] were bred into a C57/B6 background. Heterozygous-heterozygous breeding was used to generate Kv4.2-/- and wild-type (WT) littermates. Genotyping was performed using the following primers: K_V_4.2 (613 bps; forward, GTGGAT GCC TGT TGC TTC; reverse, CCC ACA AGG CAG TTC TTT TA) and aminoglycoside phosphotransferase (neomycin-resistance) (500 bps; forward, AGG ATC TCC TGT CAT CTC ACC TTGCTC CTG; reverse, AAG AAC TCG TCA AGA AGG CGA TAG AAG GCG) [15].

### Isolation of Adult Mouse Ventricular Myocytes

As previously described [13], hearts were rapidly removed from anesthetized (4% isoflurane oxygen mixture inhalation) mice and retrogradely perfused with Ca^2+^-free Tyrode’s solution, which contained (in mM): 137 NaCl, 5.4 KCl, 1.0 MgCl_2_, 0.33 NaH_2_PO_4_, 10 D-glucose, 10 HEPES, pH 7.4, at 37°C through aorta for 3–4 min. Then it was perfused with collagenase (Type II, 1.0 mg/mL, Worthington) for 10–12 min. The EPI and ENDO heart cells were collected with a sharp forceps under a dissecting microscope. After heart was digested, the right ventricle was removed and an incision was made into the ventricular septum in order to expose the endocardium (ENDO) of the left ventricular free wall. A small amount of ENDO tissue (~0.05mm^3^) was collected from left ventricular free wall inner surface at the level of the tip of papillary muscle. Then the outside surface of left ventricular was washed with Krebs bicarbonate solution and a small amount (~0.05mm^3^) of EPI tissue was picked up from the middle point of left ventricular free wall. The digested ventricular tissue was gently triturated in the cell storage solution, which contained (in mM): 120 potassium glutamate, 20 KCl, 20 HEPES, 1.0 MgCl_2_, 10 D-glucose, 0.5 K-EGTA, and 0.1% bovine serum albumin.

### Patch Clamp Electrophysiology & Data Analysis

Voltage-activated K^+^ currents from isolated ventricular myocytes were recorded with the whole-cell patch clamp technique (Axopatch 200B and Clampex8 software, Axon Instrument, CA, USA) at room temperature (24°C). Measurements were made at room temperature in order to facilitate comparisons with previous studies in which voltage-gated K^+^ currents were quantified in mouse cardiomyocytes [4, 7, 11, 16, 17] and in particular to allow comparisons with our previous study wherein a method was developed to reliably dissect voltage-gated K^+^ currents in mouse cardiomyocytes [13]. Cells were perfused with bath solution for 15 minutes before electrophysiological recording. Ca^2+^ tolerant rod-shape cardiomyocytes were selected and examined. The bath solution contained (in mM): 140 NaCl, 4 KCl, 1 MgCl_2_, 1.2 CaCl_2_, 10 HEPES, 10 D-glucose, and 0.3 CdCl_2_ (pH 7.4). The pipette resistance ranged between 1.2~2.0 MΩ when filled with a pipette solution, which contained (in mM): 120 potassium aspartate, 20 KCl, 10 NaCl, 1 MgCl_2_, 5 MgATP, 10 HEPES, and 10 EGTA (pH 7.2). Cell capacitance and series resistance were electronically compensated by 85%. Voltage-gated K^+^ currents were induced with a 25 second depolarization from a holding potential of -80 mV to +60 mV, at which membrane potential K^+^ channels are fully activated and also minimize the contributions of potential overlapping Na^+^ currents. A double-pulse protocol was used for characterizing K^+^ currents recovery from inactivation kinetics. Cells were first depolarized to +60 mV for 20 sec (pre-pulse), subsequently hyperpolarized to the holding potential at -80 mV for various times ranging from 10 ms to 6 sec, and then stepped to +60 mV for another 5–20 sec (test-pulse) to activate the currents and assess the extent of recovery; sweep interval was set at 45 sec for full recovery of all K^+^ currents and allow sufficient time to adequately check and adjust (as required) the series-resistance compensation and resting membrane potential before each subsequent depolarization. HpTx-2 (5 μM) was used in bath solution for certain experiments.

Electrophysiological data were analyzed using pClamp software (Clampfit9, Axon, CA, USA). Inactivation of voltage-dependent outward K^+^ currents was fitted with a sum of three exponentials:

$$f\left(t\right)=A_{1}e^{-t/\tau_{1}}+A_{2}e^{-t/\tau_{2}}+A_{3}e^{-t/\tau_{3}}+\text{C}$$ (1)

Here, A is the current amplitude, τ is inactivation time constant, C is non-inactivate current component (I_ss_). The goodness of fitting was evaluated by the correlation coefficients (R value).

To quantify the recovery of I_to_ in mouse cardiomyocyte, I_to_ in both pre-pulse and test-pulse were dissected from I_Kslow1_, I_Kslow2_ and I_ss_ with 3-exponential curve fitting. I_to_ in test-pulse was normalized to that in pre-pulse, and the ratio was plotted as a function of pulse interval time and fit with a biphasic equation:

$$I/I_{0}=A_{1}\left(1-e^{-\frac{t}{\tau_{1}}}\right)+A_{2}(1-e^{-\frac{t}{\tau_{2}}})$$ (2)

Here A is percentage of each component in whole current (A_1_ + A_2_ = 1), τ is the recovery time constant.

### Immunofluorescent staining

Immunofluorescent staining was performed as previously described [18]. In brief, isolated myocytes were plated on laminin-coated (0.5 mg/mL, Roche) glass coverslips, fixed with 4% paraformaldehyde in PBS for 1 hour at room temperature, and permeated with 0.2% (v/v) Triton X-100 for 10 min. After blocking with 1% bovine serum albumin in PBS, cells were exposed to primary antibody against Kv4.3 (1:100) in blocking solution overnight at 4°C. The anti-Kv4.3 mouse monoclonal antibodies (NeuroMab) were raised against a fusion protein corresponding to amino acids 415–636 in the cytoplasmic C-terminus of a rat Kv4.3 protein [19]. A secondary Alexa Fluor 488 goat anti-mouse IgG (Invitrogen) was subsequently used for visualization. Images were acquired with an argon laser beam equipped confocal microscope (excitation 488nm, emission 519nm; Olympus). To control for background and cell auto-fluorescence, parameters were normalized based on fluorescence intensities measured in control samples containing no antibody, primary antibody only or secondary antibody. The Kv4.3 expression was quantified by the average fluorescence signals in 3-dimensional reconstructions of immunofluorescence images of isolated myocytes obtained using optical Z sections (0.5 μm thickness) with a 60X magnification oil objective in conjunction with a Kalman filter (n = 3). The distribution of fluorescence density across the cells in the middle layer image was analyzed. 3D construction was performed using ImagePro Plus (Media Cybernetcis).

### Quantitative RT-PCR

Total RNA was isolated with TRIZOL reagent (Life Technologies), and then cDNA was synthesized from 1.0 μg of RNA using Superscript II reverse transcriptase (Life Technologies). The samples from different regions of the heart (i.e. left ventricular epicardium and endocardium) were obtained by dissection from the left ventricular free wall using a scalpel with the aid of a dissecting microscope. Gene expression assay was conducted on 10 ng of template cDNA by Quantitative PCR (qPCR) using Taqman and SYBR green PCR methods equipped with ABI 7900HT (Applied Biosystems). Primers for mouse *Kcnd2* (Kv4.2), *Kcnd3* (Kv4.3) and *Kcnip2* (KChIP2) are as follows: *Kcnd2* forward, 5’-GTGTCGGGAAGCCATAGAGGC-3’, Reverse, 5’-TTACAAGGCAGACACCCTGA-3’; *Kcnd3* forward, 5’-CTCCCGTCGTAGCAAGAAGA-3’, Reverse, 5’-GGTGGGGATGCTGATGATG-3’; and *Kcnip2* forward, 5’-GGCTGTATCACGAAGGAGGAA-3’, Reverse, 5’- CCGTCCTTGTTTCTGTCCATC-3’. The relative expression of these genes was determined using the normalized comparative CT method [20] with *Gapdh* as the background gene, which did not vary between regions. The total cycle numbers for all experiments was 35 and the products were verified using both melt tests and agarose gels. Thresholds were determined from the linear regions of the amplification curves and the efficiency of the probes was estimated using serial dilutions.

### Statistics

All results were expressed as Mean ± S.E.M. Statistical significance of differences were determined by Student’s *t* test (paired or unpaired) and ANOVA. Differences at *P*< 0.05 were considered statistically significant. Calculations and statistical tests were performed using Prism5.03 (GraphPad Software, San Diego, CA, USA).

## Results

### I_to, f_ is present in Kv4.2-/- endocardial myocytes (ENDO)

**Fig 1:**
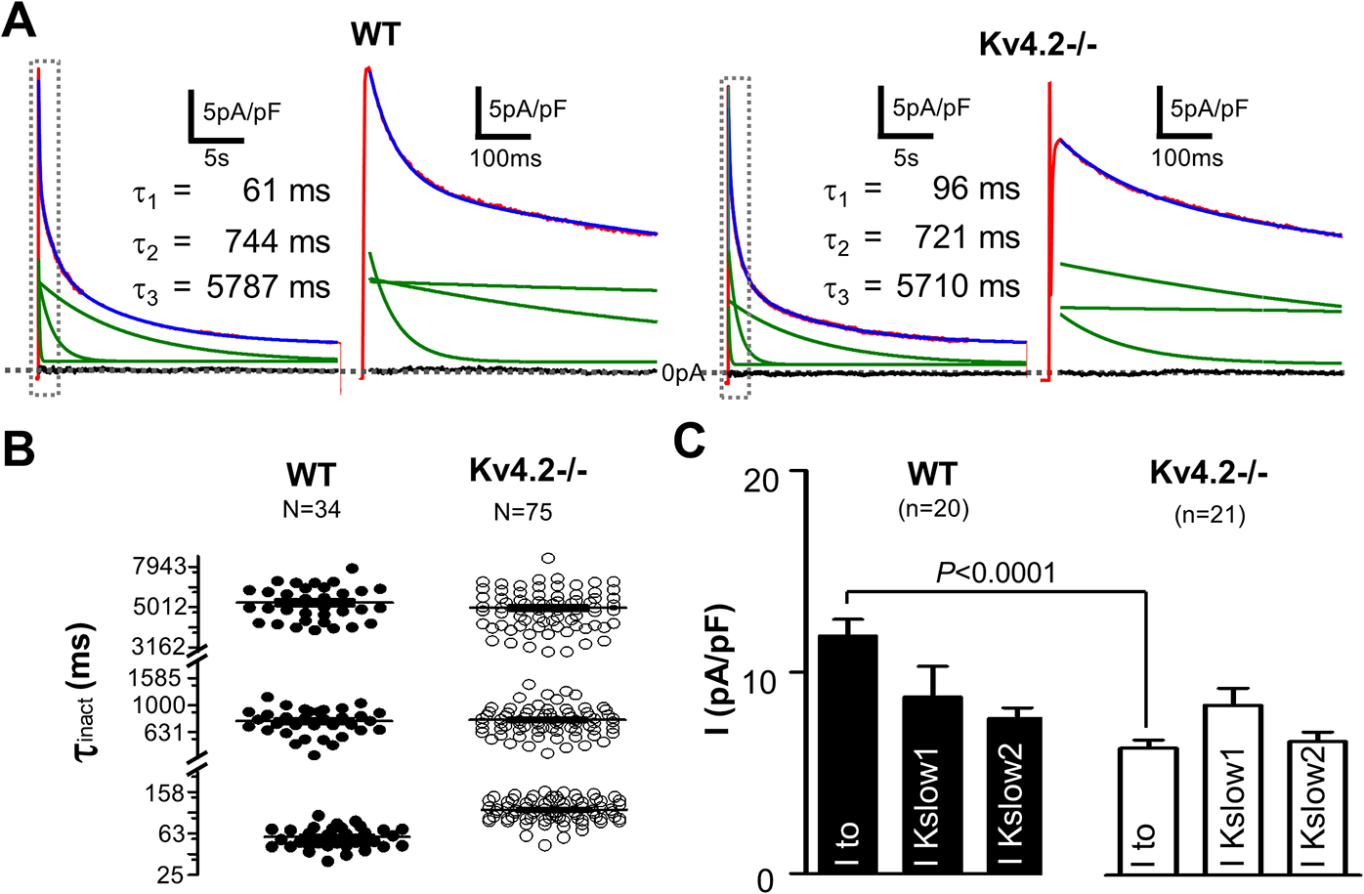
Outward K^+^ currents measured in ENDO cardiomyocytes isolated from WT and Kv4.2 -/- hearts. (A) Typical outward K^**+**^ **c**urrents measured in both the WT and Kv4.2-/- are shown following step depolarization to +60mV (for 25s) from a holding potential of -80mV. The 2^**nd**^ and 4^**th**^ panels are 50-folds time expanded relative to the area in the boxes on their left side adjacent panels (1^**st**^ and 3^**rd**^ panels). Voltage-dependent K^**+**^ currents recorded from both groups of cardiomyocytes could be well fit with tri-exponential functions (show in blue). The three green lines in each panel represent the three individual components of the tri-exponential functions, and their time constant (t) are shown in the inset tables. The residuals (i.e. the differences between the red and blue curves) are also shown in black and, as can be seen, these residuals are essentially zero consistent with the very high correlation coefficients (R-values) typically estimated in the fits of all our traces (which ranged from 0.976–0.995 with 88% of R-values being > 0.98). (B) Summary of the predicted inactivation time constants of the three individual exponential components of the tri-exponential functions that provided the best fit for all K^**+**^ current traces analyzed. These three components were labeled based on their kinetics as: I_Kslow2_ (slowest), I_Kslow1_ (intermediate) and I_to_ (fastest). We previously demonstrated that tri-exponential fits provide optimal fits to the outward K^**+**^ currents thereby allowing the different underlying current to be quantified in detail^**21**^. Notice that the kinetics (i.e. the τ value) of I_Kslow2_ and I_Kslow1_ are unchanged while kinetics of I_to_ are slower (*P*<0.0001) in Kv4.2-/- than WT. This shift in the kinetics of I_to_ is explained and discussed further in the text. C) Shows the current densities for I_Kslow2_, I_Kslow1_ and I_to_ in WT and Kv4.2-/- cardiomyocytes estimated from the amplitudes of the three kinetic K^**+**^ current components (obtained from the fits to the individual current traces). As can be seen that I_to_ density was much smaller, as expected, in Kv4.2-/- compared to WT while I_K,slow2_ and I_K,slow1_ are comparable between WT and Kv4.2-/-.

**Fig 2:**
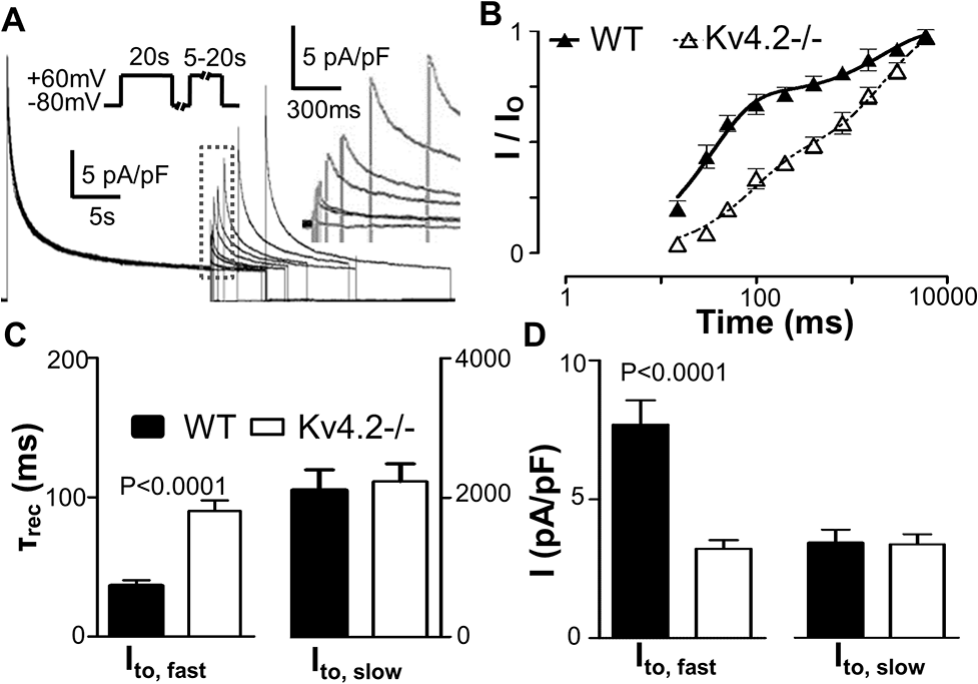
Recovery from inactivation kinetics of I_to_ (i.e. the fastest inactivation component of the outward K^+^ currents) in WT and Kv4.2-/-cardiomyocyte. (A) Shows representative K^**+**^ current recovery from inactivation recordings from a typical Kv4.2-/- cardiomyocyte. See Methods for the specific protocol. The early portions of traces following the depolarizing test-pulse are re-shown on a faster (expanded to 300 ms/bar scale) time scale in the inset (broken box) to better illustrate the properties of the time-course (of the recovery) of the peaks of the I_to_ K^**+**^ currents as a result of membrane repolarization (to -80mV). (B) This panel shows the normalized I_to_ current as a function of the pulse interval duration during the recovery from inactivation protocol. The normalized current is defined as the amplitude of the I_to_ density (i.e. the fastest component) estimated from fits to the currents elicited following depolarization during the recovery period (as described in Fig 1) divided by the I_to_ density (i.e. fastest component) estimated from fits of the current measured following depolarization during the initial conditioning pulse. Notice that the time course of the recovery of the I_to_ current shows clear bi-phasic properties, as we described previously, in ENDO cardiomyocytes from WT and Kv4.2-/- hearts. This bi-phasic recovery property of I_to_ clearly indicates that I_to_ induced following depolarization is comprised of two underlying currents with different recovery kinetics, a fast recovery component (I_to,f_) and a slow component (I_to,s_). (C) This shows a summary of the kinetics of the biphasic recovery (i.e. τ_rec_) allowing separation into I_to,f_ and I_to,s_. Note that the τ_rec_ for I_to,f_ is increased and while that for I_to,s_ is unchanged in Kv4.2-/- compared to WT. (D) Shows the estimated current density of I_to, f_ and I_to, s_ calculated from the relative proportions of the I_to_ recovering quickly or slowly from inactivation, respectively. Note that Kv4.2-/- decreases I_to,f_ but not I_to, s_.

Voltage-dependent K+ currents in mouse cardiomyocytes are generated by Kv4.2/Kv4.3, Kv1.4, Kv1.5 and Kv2.1-encoded channels [4, 14, 16, 17, 21–27]. Currents generated by Kv4.2/Kv4.3 and Kv1.4 channels have similar activation and inactivation kinetics (fast), with inactivation time constants (τ_inact_) < 150 ms; while the τ_inact_ of Kv1.5 and Kv2.1 channels are ~800 ms (intermediate) and ~4500 ms (slow), respectively. Based on these biophysics features, previous studies have established that voltage-gated K^+^ currents, when stimulated from rest with depolarizing pulses lasting 25 sec, are best described with 3-exponential function and four temporal components are dissected: a sustained component (i.e. I_sus_), a “slow” component (i.e. I_K,slow2_ probably encoded by Kv2.1), an “intermediate” component (i.e. I_K,slow1_ probably encoded by Kv1.5), and a “fast” component (i.e. I_to_) which can be further separated, using recovery from inactivation protocols based on their significantly different recovery kinetics, into an I_to,f_ (encoded by Kv4.2/3) and an I_to,s_ (encoded by Kv1.4-encoded) component [13]. Fig 1A shows K^+^ currents recorded using 25 sec depolarizing pulses (to +60mV) from a holding potential of -80mV recorded in wild-type (WT) and Kv4.2-/- cardiomyocytes. As reported previously, the outward K^+^ currents in WT cardiomyocytes (isolated from the ENDO) are well fit with tri-exponential functions (R-value range was 0.976–0.995, 88% of R-values were > 0.98). Cardiomyocytes from Kv4.2-/- mouse hearts could be similarly fit well with tri-exponential functions and we found no differences in either the amplitudes or decay (i.e. inactivation) kinetics of the I_k,slow1_, I_k,slow2_ and I_sus_ components between the groups (Fig 1B and Table 1). On the other hand, the current density of the fastest decaying component (i.e. I_to_) was reduced (*P*<0.0001) in Kv4.2-/- ENDO myocytes (6.89 ± 0.44 pA/pF, n = 21) compared to WT (11.80 ± 0.82 pA/pF, n = 20) (Fig 1C and Table 1). Recovery from inactivation of the fastest decaying component (i.e. I_to_) yields a bi-exponential time course (Fig 2 and Table 2) with the fast recovering portion (i.e. I_to,f_) having a lower (*P*<0.0001) density in Kv4.2-/- ENDO cardiomyocytes (3.21 ± 0.31 pA/pF, n = 12) compared to WT (7.69 ± 0.87 pA/pF, n = 12) without differences (*P* = 0.9111) in slower component (i.e. I_to,s_). These findings support the conclusion that I_to,f_ is present in Kv4.2-/- cardiomyocytes but at reduced levels compared to WT without changes in the other voltage-gated K^+^ currents in ENDO myocyte. It is worthy note that the estimated rate of decay (i.e. τ_inact_) of the fastest component (i.e. I_to_ = I_to,f_ + I_to,s_) was faster (*P*<0.0001) in WT cardiomyocytes (58 ± 2 ms, n = 34) than in Kv4.2-/- cardiomyocytes (106 ± 3 ms, n = 75) which parallels differences in inactivation kinetics between Kv4.2 versus Kv4.3 currents in heterologous expression systems [22, 28, 29], as discussed further below.

**Table 1:** Comparison of potassium current density and relative amplitude in ventricular myocyte.

		I_to_	I_Kslow1_	I_Kslow2_	I_sus_
**ENDO**					
**WT**	Density (pA/pF)	11.80±0.82	8.78±1.51	7.71±0.52	4.67±0.34
n = 20	Amplitude (%)	36±0.31	27±0.62	24±0.54	13±0.29
**Kv4.2-/-**	Density (pA/pF)	6.89±0.44*	9.23±0.91	7.25±0.51	4.02±0.55
n = 21	Amplitude (%)	24±0.27	33±0.42	26±0.37	17±0.41
**EPI**					
**WT**	Density (pA/pF)	28.37±1.21*	33.45±2.95*	18.52±1.77*	7.33±0.57*
n = 14	Amplitude (%)	32±0.86	38±0.40	21±0.96	9±0.37
**Kv4.2-/-**	Density (pA/pF)	8.96±0.78^ #	31.12±1.88^ #	10.98±0.63^ #	4.39±0.28^
n = 54	Amplitude (%)	17±0.51	56±0.45	20±0.84	7±0.23

* P<0.05, compared to WT ENDO

^ P<0.05, compared to WT EPI

# P<0.05, compared to Kv4.2-/- ENDO

**Table 2:** Comparison of I_to_ recovery from inactivation in ventricular myocytes.

		I_to,f_	I_to,s_
**ENDO without HpTx-2**			
**WT**	τ_rec_ (ms)	33±3	2256±292
n = 12	Density (pA/pF)	7.69 ± 0.87	3.78±0.50
	Amplitude (%)	67±9	33±9
**Kv4.2-/-**	τ_rec_ (ms)	88±10^	2267±217
n = 12	Density (pA/pF)	3.21±0.31^ #	3.71±0.39
	Amplitude (%)	45±7	55±7
**ENDO with HpTx-2 (5 μM)**			
**WT**	τ_rec_ (ms)		1906±263
n = 6	Density (pA/pF)		4.36±0.93
**Kv4.2-/-**	τ_rec_ (ms)		2050±190
n = 6	Density (pA/pF)		4.13±0.75
**EPI without HpTx-2**			
**WT**	τ_rec_ (ms)	32±4 #	2187±277
n = 12	Density (pA/pF)	27.46±1.38^ #	0.63± 0.40^ #
	Amplitude (%)	98±1	2±1
**Kv4.2-/-**	τ_rec_ (ms)	86±4	2227±310
n = 12	Density (pA/pF)	4.64±0.28	3.92±0.41
	Amplitude (%)	56±7	44±7

^: P<0.05 compared to WT ENDO

#: P<0.05 compared to Kv4.2-/- EPI.

**Fig 3:**
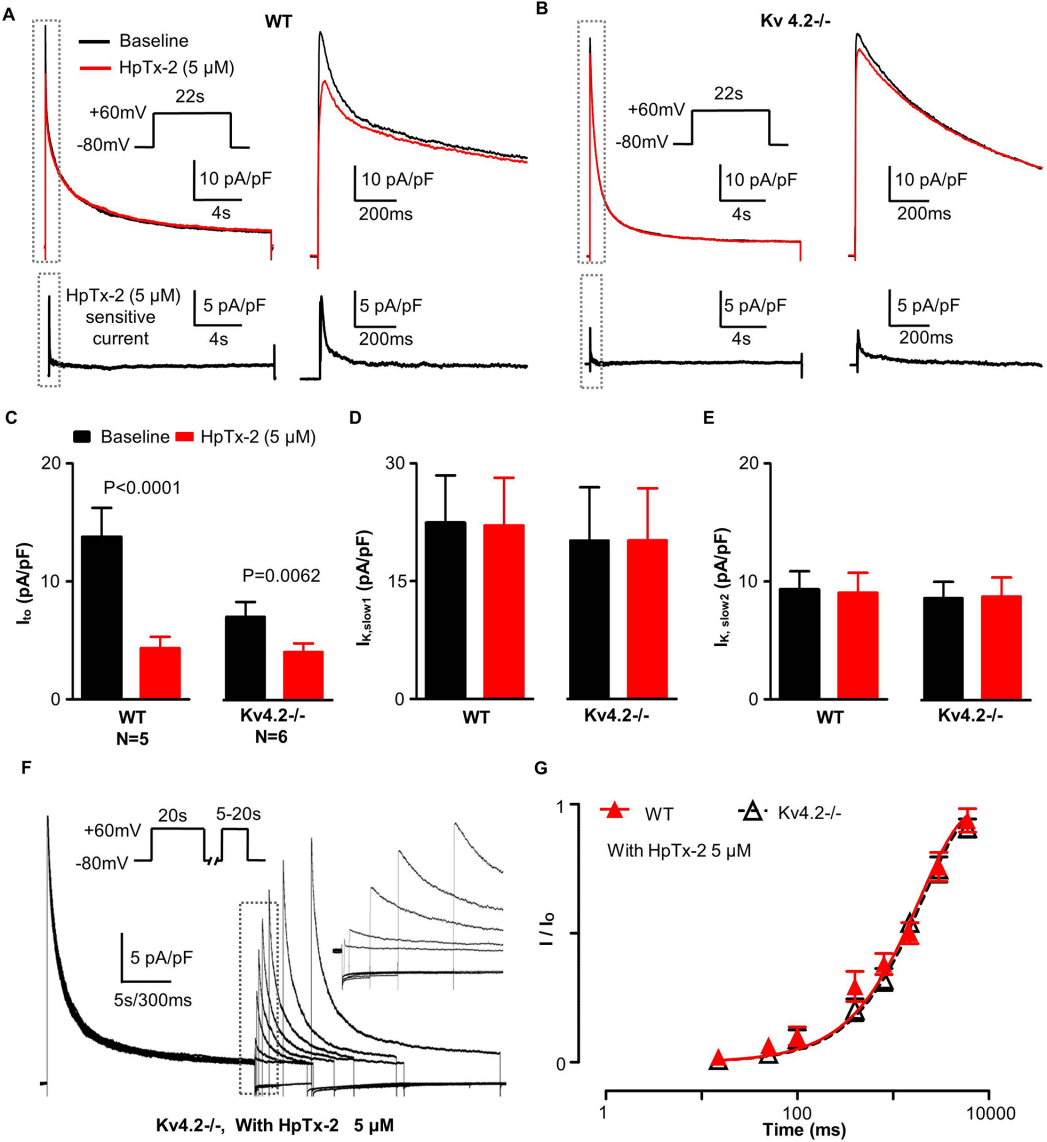
Effect of HpTx-2 on K^+^ current from isolated WT and Kv4.2-/- cardiomyocyte. (A) This panel illustrates representative K^**+**^ current traces recorded from isolated WT cardiomyocyte under baseline (dark) conditions and after the addition of 5 μM HpTx-2 (red), a high-selective Kv4.x channel blocker. The HpTx-2 sensitive current was obtained by arithmetic subtraction of the red trace (with HpTx-2) from the dark trace (baseline) control and is shown in the lower trace. The initial parts of traces (gray dashed box) are shown on a more rapid time scale on their right side. (B) Shows representative K^**+**^ current traces from a Kv4.2-/- cardiomyocyte under baseline (dark) conditions and after the addition of 5 μM HpTx-2 (red). The HpTx-2 sensitive currents are shown in the lower trace. (C) Show here is the I_to_ current density estimated under both baseline conditions and after HpTx-2 treatment from the tri-exponential curve fits to the K^**+**^ currents recorded (as described in Fig 1). Note that while HpTx-2 decreased the I_to_ current in both WT and Kv4.2-/-, the decrease was far greater (*P*<0.01) in WT than the Kv4.2-/- cardiomyocytes. (D) and (E) These panels show I_K, slow1_ and I_K, slow2_ current density estimated (using the curve fitting methods) under baseline conditions and after HpTx-2 addition. After HpTx-2 these two currents density remained fixed and did not differ between WT and Kv4.2-/- cardiomyocytes. (F) Shows representative K^**+**^ current recovery from inactivation recordings from a typical Kv4.2-/- cardiomyocyte with 5 μM HpTx-2. See Methods for the specific protocol. The early portions of traces following the depolarizing test-pulse are re-shown on a faster (expanded to 300 ms/bar scale) time scale in the inset (broken box) to better illustrate the properties of the time-course (of the recovery) of the peaks of the I_to_ K^**+**^ currents as a result of membrane repolarization (to -80mV). (G) This panel shows the normalized I_to_ current as a function of the pulse interval duration during the recovery from inactivation protocol with 5 μM HpTx-2. The normalized current is defined as the amplitude of the I_to_ density (i.e. the fastest component) estimated from fits to the currents elicited following depolarization during the recovery period (as described in Fig 1) divided by the I_to_ density (i.e. fastest component) estimated from fits of the current measured following depolarization during the initial conditioning pulse. Notice that, the recovery of I_to_ in both WT and Kv4.2-/- here are similar and are best fit with mono-exponential equation without the fast recovery component (that is, I_to,f_) showed in Fig 2B. Moreover, they were comparable to the slow components (that is, I_to,s_) dissected in Fig 2B without HpTx-2.

To confirm the presence of I_to,f_ in Kv4.2-/- myocytes, we applied the specific blocker of Kv4.2/3 channels, HpTx-2. As showed in Fig 3, HpTx-2 (5 μM) only affected K^+^ currents in first ~400 ms following the depolarization and reduced (*P* = 0.0062) the fast component of the voltage-gated K^+^ currents (i.e. I_to_) in ENDO cardiomyocytes by 3.0 3± 0.67 pA/pF (n = 6) in Kv4.2-/- cardiomyocytes (Fig 3), without affecting I_to,s_, I_k,slow1,_ I_k,slow2_ or I_sus_.This extent of I_to_ reduction is remarkably similar to (*P* = 0.7841) to the I_to,f_ density estimated from fits to the fastest kinetic component in recovery from inactivation voltage protocols (i.e. 3.21 ± 0.31 pA/pF), as summarized in Table 2. In addition, the decay kinetics (τ_inact_) of HpTx-2-sensitive current displayed a mono-exponential time course in both groups with τ_inact_ being larger (*P*<0.0001) in Kv4.2-/- cardiomyocytes (85 ± 11 ms, n = 6) than in WT (53 ± 6 ms, n = 5). Consistent with these results, HpTx-2 blocked the fast recovery component (i.e. I_to,f_) in both WT and Kv4.2-/- cardiomyocytes without affecting the slow component (I_to,s_). Specifically, as summarized in Table 2, I_to_ recovery from inactivation in Kv4.2-/- followed a mono-exponential time course in the presence of 5 μM HpTx-2 with a time constant (τ_rec_ = 2050 ± 190 ms, n = 6, R = 0.9939) which was indistinguishable (*P* = 0.8112) to the estimated τ_rec_ for I_to,s_ (2267 ± 217 ms, n = 12, R = 0.9950) when HpTx-2 was absent. Similarly, in WT cardiomyocytes, τ_rec_ was estimated to be 1906 ± 263 ms (n = 6, R = 0.9862) in the presence HpTx-2 which did not differ (*P* = 0.7769) from τ_rec_ for the slow recovering component (2256 ± 292 ms, n = 12, R = 0.9878) without HpTx-2. Finally, no differences in τ_rec_ of the slow recovering components of I_to_ were observed between Kv4.2-/- and WT with (*P* = 0.6214) or without (*P* = 0.8611) HpTx-2 (Table 2).

### Expression of Kv4.3 α-subunit at the plasma membrane of myocytes

**Fig 4:**
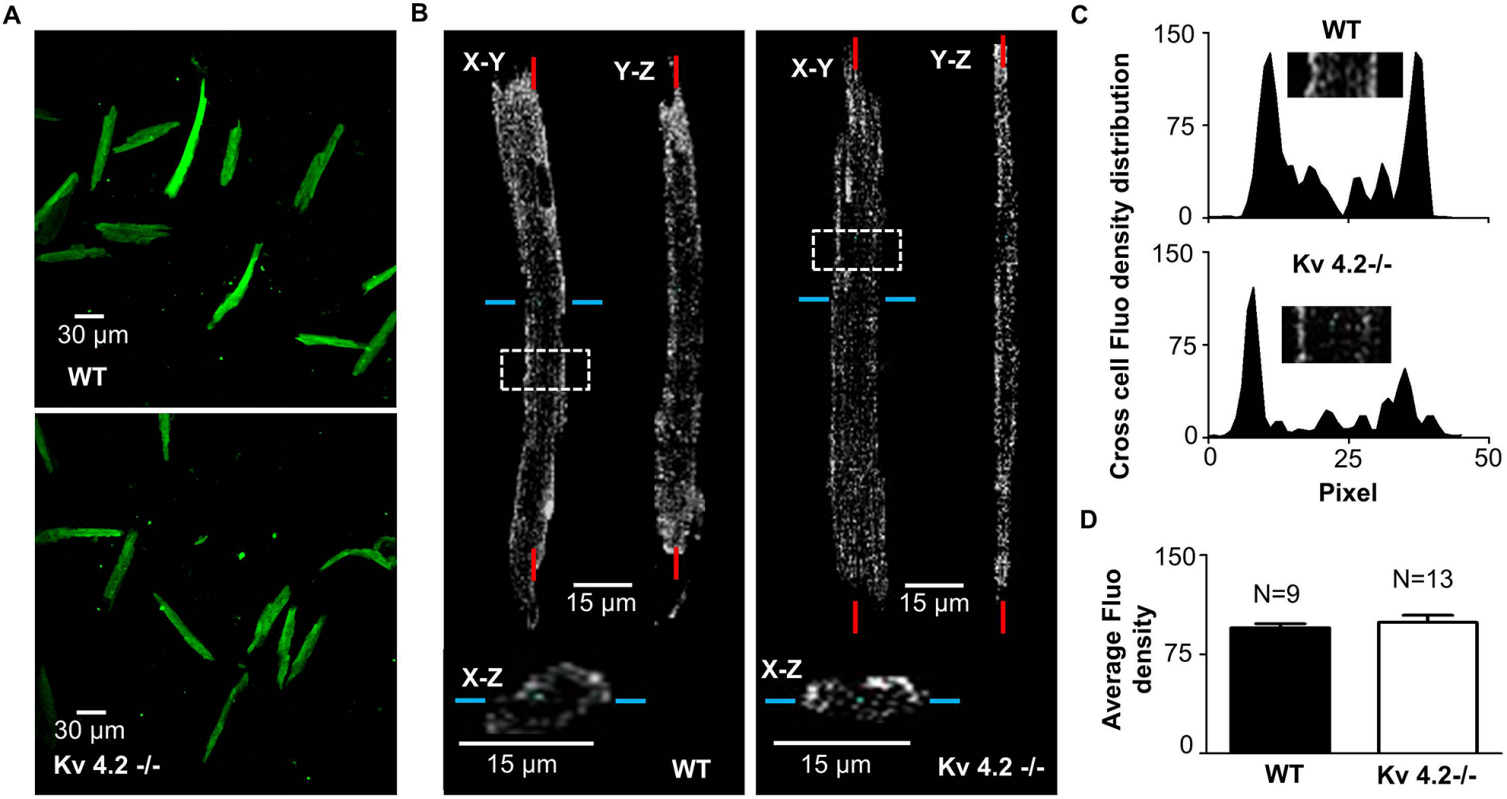
Membrane expression of Kv4.3 subunit in isolated WT and Kv4.2-/- cardiomyocyte. (A) Adult WT (top panel) and Kv4.2-/- (bottom panel) mouse ventricular cardiac myocytes labeled with anti-Kv4.3 antibody show positive fluorescent staining (originating from the 2^**nd**^antibody) at 10X magnification. (B) This panel shows the 3D reconstruction of a series of Z-stack fluorescent images of a typical cardiomyocyte taken using a confocal microscope. In the upper part of the figure the fluorescence pattern is shown for cardiomyocytes in two longitudinal orientations (X-Y and Y-Z planes). A cross-section was take through the cardiomyocytes at the position indicated by the blue line and is shown in the bottom (the X-Z plane). The results clearly demonstrate that the fluorescent signals originate primarily from the regions at the periphery for both WT (left panel) and Kv4.2-/- (right panel) cardiomyocytes. (C) This shows the fluorescent density distribution across the WT and a Kv4.2-/- cardiomyocyte shown in Panel B in the regions identified by the white boxes. (D) This quantifies the average integrated fluorescence density for cardiomocytes from WT (n = 9 cardiomyocytes from 4 hearts) and Kv4.2-/- (n = 13 cardiomyocytes from 6 heart) hearts. Average fluorescent density was calculated by summing the total fluorescent signal intensity normalized for the total number of pixels for each cardiomyocyte. Note this quantifies and the Kv4.3-subunit expression level in each cardiomyocyte.

Although as expected Kv4.2 protein was not detected in Kv4.2-/- myocardium[11], Kv4.3 protein is expressed in Kv4.2-/- myocardium even though I_to,f_ was failed to be detected using the short depolarization pulses (lasting ~5 sec) method[11], and these observations were considered as key to conclusion that Kv4.2 is required for functional integration of Kv4.3-based channels into the cardiomyocyte membrane[11]. However, in heterologous expression systems Kv4.3 alone generates I_to,f_ and is inserted into cell membrane[23, 30]. We therefore performed immunofluorescent staining with Kv4.3 antibodies. As can be seen, from longitudinal and transverse three-dimensional reconstructions of isolated cells labeled with Kv4.3 antibodies (with the appropriate secondary antibodies), Kv4.3 α-subunits are clearly present at the membrane as indicated by the higher fluorescent density at the periphery of the cell than cell interior in both WT and Kv4.2-/- ventricular myocytes (Fig 4). The average fluorescent density in the periphery were comparable (*P* = 0.4886) between WT (99.17 ± 5.32, n = 9) and Kv4.2-/- (95.20 ± 2.91, n = 13).

### I_to_ heterogeneity in the ventricular free wall of Kv4.2-/-

**Fig 5:**
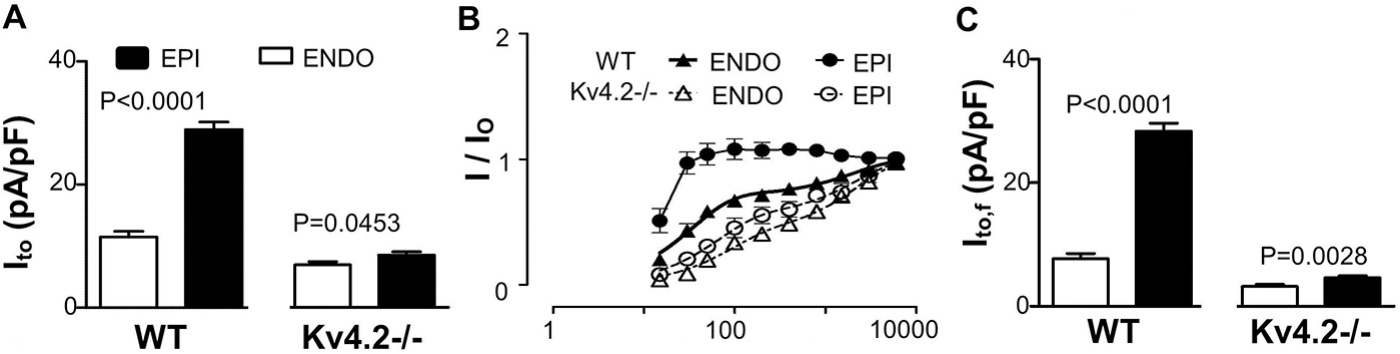
Heterogeneity of I_to_ and I_to,f_ in left ventricular free wall cardiomyocytes in WT and Kv4.2-/-. (A) This shows the average I_to_ density (I_to_ = I_to,f_ + I_to,s_) estimated in EPI and ENDO cardiomyocytes isolated from WT and Kv4.2-/- hearts (n = 12 for all groups) using the tri-exponential fitting protocol (described in Fig 1). As we can see, I_to_ density in Kv4.2-/- was reduced compared to WT for both ENDO and EPI cardiomyocytes. What is striking here is that the transmural gradients of I_to_ (EPI/ENDO) exist in left ventricular free walls for both WT and Kv4.2-/- but this gradient is markedly reduced in the Kv4.2-/- compared to WT. (B) Again I_to_ recovery from inactivation curve (n = 12 in each group) was used to separate I_to,f_ and I_to,s_ in I_to_ using the method described in Fig 2. (C) This panel quantifies the I_to,f_ measured in ENDO and EPI cardiomyocytes from WT and Kv4.2-/- hearts (n = 6 for all groups).

**Fig 6:**
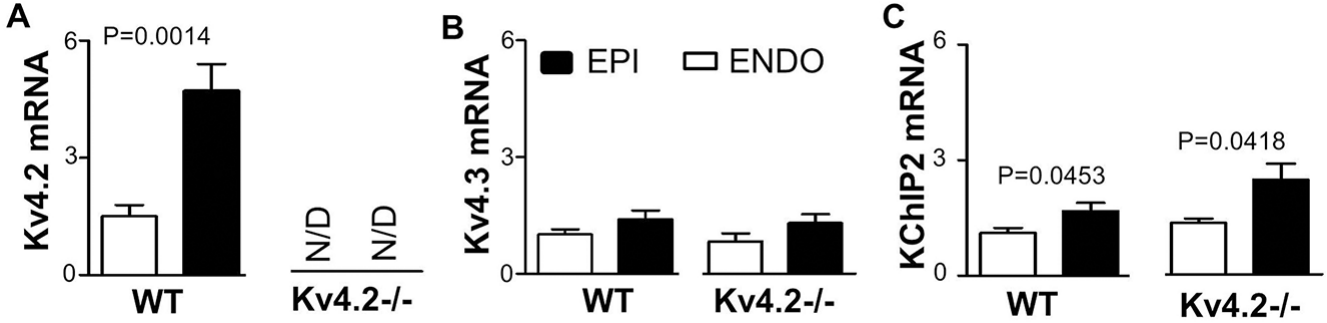
Kv4.2, Kv4.3 and KChIP2 mRNA expression level in ENDO and EPI tissue sample from left ventricular free wall of WT and Kv4.2-/- hearts. Relative gene expression of voltage-gated K^**+**^ channels in ENDO and EPI tissues of WT (n = 6) and Kv4.2-/- (n = 6) hearts were measured by qtPCR and analyzed using the ∆∆Ct method with normalization to GAPDH, which did not change between samples. The qtPCR protocols involved 35 cycles for all reactions. All the primers were tested with by generating standard curves to measure probe efficiency and both meting curves and electrophoresis were utilized to confirm single PCR products. (A) This panel shows the estimated Kv4.2 mRNA expression levels detected in WT hearts. No specific Kv4.2 PCR product was detected in Kv4.2-/- hearts after 35 cycles (i.e. not detected, ND). Note the large EPI to ENDO gradients in the WT hearts, as expected. (B) Kv4.3 mRNA expression in both WT and Kv4.2-/- hearts showing no measureable gradient, although trends existing with more in the EPI than ENDO. (C) KChIP2 mRNA showing transmural heterogeneity (EPI>ENDO) in both the WT and Kv4.2-/- hearts.

The results above support the conclusion that Kv4.3 α-subunits can form homotetrameric functional channels and generate I_to,f_ in mouse cardiomyocytes when Kv4.2 is absent. Since the transmural I_to,f_ gradients in mouse myocardium have been linked to variations in the expression of both Kv4.2 and KChIP2 [10], we further assessed regional variations of I_to,f_ in the Kv4.2-/- hearts. In order to evaluate transmural heterogeneity of I_to,f_, K^+^ currents were recorded from epicardial (EPI) cardiomyocytes from the left ventricular free wall. The analysis revealed, that the density of I_to_ was larger in the EPI cardiomyocytes than ENDO cardiomyocytes in both WT and Kv4.2-/- hearts (Fig 5A and Table 1). More importantly, this transmural I_to_ pattern was entirely due to differences in the fast components of I_to,f_ (Table 2). After separating I_to,f_ and I_to,s_ as shown above in Fig 5B, I_to,f_ density in EPI was ~4-fold bigger than in ENDO in WT, while it only ~1.2-fold bigger in EPI than ENDO in Kv4.2-/- (Fig 5C). Thus, while Kv4.2 appears to be a major determinant of the I_to,f_ transmural gradient in mouse myocardium, a residual transmural I_to,f_ gradient remains in Kv4.2-/- and is associated with a corresponding the gradient (*P* = 0.0418) of KChIP2, but not (*P* = 0.7214) Kv4.3, mRNA expression. Interestingly, the transmural Kv4.3 mRNA gradient in the Kv4.2-/- hearts did not differ (*P* = 0.8633) from that in the WT hearts (Fig 6).

## Discussion

Our results establish that, when long depolarization pulses (25 sec), I_to,f_ can be readily identified. This conclusion clearly contrasts with a previous study [11] concluding that I_to,f_ is absent in Kv4.2-/- hearts. We believe the lack of agreement can be readily explained by differences in the voltage protocols and methods of quantifying the time dependent K^+^ current traces. Specifically, in a previous study [13] we showed that, when K^+^ current traces are lasting ~25 s, K^+^ current traces are best fit with tri-exponential functions. This conclusion can be readily illustrated by showing that the distribution of the τ_inact_ values obtained from tri-exponential fits are much “tighter” (i.e. smaller variance) than when either bi-exponential or quadri-exponential fits to the traces were used [13]. Our results further showed that the estimates of the magnitude and time constants of the different current components become increasingly inaccurate as the length of the traces (used for the fits) are shortened. These observations are consistent with our analysis showing that tri-exponential functions gave the best statistical fits to the K^+^ current traces [31]. We confirmed these findings in the Kv4.2-/- cardiomyocytes (data not shown) and found further that only the component with fastest time constant (i.e. τ_inact_ for I_to_) differed with WT cardiomyocytes. One potential complication when using long depolarization pulses is K^+^ ion depletion which could impact on the measured kinetics of K^+^ current decay during the depolarizing pulses. However, the resting membrane potentials did not differ immediately before and after the long depolarizing pulses (i.e. before -72.38 ± 0.29 mV, n = 200 vs. after -72.38 ± 0.29 mV, n = 200; S1 Table). These observations are not unexpected since the amount of K^+^ that is able to diffuse from the pipette (1.2~ 2 MΩ, 1~3 μm tip) into the cell is predicted, from the application of Fick's Law of Diffusion [32, 33], to be at least 10-fold greater than the amount of K^+^ that leaves the cardiomyocytes through K^+^ channels (i.e. 2.92 ± 0.09 × 10^−7^ Coulombs) during the depolarizing pulses under our experimental conditions. We are confident that the long depolarization pulses did not significantly distort the various K^+^ currents or the conclusions derived from our analysis.

Although, as expected, the estimated density of I_to,f_ was reduced in the Kv4.2-/- cardiomyocytes compared to WT, there were also notable difference in both the kinetics of inactivation (τ_inact_) and recovery from inactivation (τ_rec_) of I_to,f_ between cardiomyocytes from Kv4.2-/- (τ_inact_ = ~105ms and τ_tec_ = ~87ms) versus for WT (τ_inact_ = ~58ms and τ_rec_ *=* ~34ms) hearts. The deceleration in the rates of inactivation and recovery from inactivation observed in Kv4.2-/- cardiomyocytes are consistent with slower intrinsic inactivation properties of Kv4.3-based currents compared to Kv4.2-based currents observed in heterologous expression systems [28]. Taken together, these results support the conclusion that I_to,f_ in mouse cardiomyocytes can be formed by Kv4.3 alone, and does not require co-assembly of Kv4.2 and Kv4.3 [11]. This conclusion is further supported by our toxin studies showing that the highly selective blocker of Kv4 currents, HpTx-2, was reduced K^+^ currents in Kv4.2-/- cardiomyocyte during the first 400 ms after depolarization only, which is the period during which I_to,f_ is expected to be active (i.e. not inactivated). Moreover, the densities of the HpTx-2-sensitive current were indistinguishable from the I_to,f_ estimated from the analysis of our curve fits to the voltage-gated K^+^ currents. In addition, the HpTx-2-sensitive currents in Kv4.2-/- cardiomyocytes had decay properties that were also slower than in WT cardiomyocytes, as expected if I_to,f_ in the Kv4.2-/- cardiomyocytes is comprised of Kv4.3-based channels [28]. HpTx-2 also eliminated the fast recovering component of I_to_ in our recover from inactivation measurements (i.e. I_to,f_) in both WT and Kv4.2-/-, which uncovered mono-exponential slow recovering component (i.e. I_to,s_) in both groups with identical magnitudes and kinetics. Our conclusion that I_to,f_ does not require Kv4.2/3 co-assembly in mouse myocardium is supported by a recent study showing that I_to,f_ is detectable in cardiomyocytes from mice lacking Kv4.3, which showed accelerated rates of inactivation compared to wild-type [12]. This conclusion is also consistent with the observation in Western Blots that the Kv4.3 protein (and mRNA) are present in Kv4.2-/- hearts at levels equivalent to that in WT hearts [11]. We confirmed that no significant differences in either Kv4.3 mRNA expression and or Kv4.3 protein in the membranes as measured immunohistochemically between Kv4.2-/- and WT cardiomyocytes.

Another novel finding of our studies is the identification of a transmural gradient in the Kv4.2-/- hearts. Previous studies have established that the gradients of I_to,f_ underlie regional differences in the amplitude and kinetics of contraction in rodent [34, 35] and dog [36] hearts, which are predicted to synchronize contraction across the ventricular wall [1]. Regardless, the I_to,f_ gradient in the Kv4.2-/- hearts was associated with a gradient in KChIP2, as we can see its expression seems increase a little in Kv4.2-/- in Fig 6, but not Kv4.3 expression. These observations suggest that the KChIP2 gradient can contribute to the transmural I_to,f_ gradient in mouse hearts, as appears to be the case in dogs [9] and humans [37] which both do not express Kv4.2 [24, 29]. However, a recent study has linked Kv4.2 mutations to cardiac arrhythmias [38]. Consistent with previous mouse studies [10] and unlike dog hearts [37], we did not see a transmural gradient of Kv4.3 expression in the mouse heart, although a trend (*P* = 0.1984) did exist for a gradient. Taken together, the observation that the transmural I_to,f_ gradient exists, albeit with a reduced magnitude, in Kv4.2-/- hearts compared to WT hearts, supports the conclusion that both Kv4.2 and KChIP2 contribute to the I_to,f_ gradient in the mouse heart.

Since the Kv4.2-/- heart expresses Kv4.3-dependent I_to,f_ like in humans cardiomyocytes, these mice might be useful for studying the regulation and consequences of I_to,f_ in humans who do not appear to express Kv4.2, although Kv4.2 mutation was associated with a single patient with J-wave syndrome [38]. This suggestion is bolstered by the observation that I_to,f_ levels and I_to,f_ channel gene expression change similarly between humans and mice in both heart disease and during heart development [3, 39–41]. Moreover, despite the fact that the action potentials durations may differ by ~10-fold between these species, the effects of changes in I_to,f_ are primarily limited to the early notch of the action potential in human cardiomyocyte which are predicted to be associated with alterations in I_Ca,L_ current profiles and thereby excitation-contraction coupling as seen in mice and other species [1, 42–46]. Future studies will be required to assess whether the Kv4.2-/- mice are a useful model for further understanding I_to,f_ in humans, and how the change of I_to,f_ affect the expression and function of Kv1.4, Kv2.1 and Kv1.5-encoded channels and currents.

## Supporting Information

S1 Table Effect of 25 s stimulation pulse on resting potential in cardiomyocytes. Resting potential in Kv4.2-/- and WT ventricular myocyte were measured with whole-cell patch-clamp technique before and immediately after 25s stimulation pulse at +60 mV. The resting potential did not changed immediately after stimulation pulse which suggests no significant intracellular K^+^ concentration changed by the stimulation pulse.
(XLSX)
